# The G‐protein biased partial κ opioid receptor agonist 6′‐GNTI blocks hippocampal paroxysmal discharges without inducing aversion

**DOI:** 10.1111/bph.13474

**Published:** 2016-04-21

**Authors:** Luca Zangrandi, Johannes Burtscher, James P MacKay, William F Colmers, Christoph Schwarzer

**Affiliations:** ^1^ Department of PharmacologyMedical University of InnsbruckInnsbruckAustria; ^2^ Department of PharmacologyUniversity of AlbertaEdmontonABCanada

## Abstract

**Background and Purpose:**

With a prevalence of 1–2%, epilepsies belong to the most frequent neurological diseases worldwide. Although antiepileptic drugs are available since several decades, the incidence of patients that are refractory to medication is still over 30%. Antiepileptic effects of κ opioid receptor (κ receptor) agonists have been proposed since the 1980s. However, their clinical use was hampered by dysphoric side effects. Recently, G‐protein biased κ receptor agonists were developed, suggesting reduced aversive effects.

**Experimental Approach:**

We investigated the effects of the κ receptor agonist U‐50488H and the G‐protein biased partial κ receptor agonist 6′‐GNTI in models of acute seizures and drug‐resistant temporal lobe epilepsy and in the conditioned place avoidance (CPA) test. Moreover, we performed slice electrophysiology to understand the functional mechanisms of 6′‐GNTI.

**Key Results:**

As previously shown for U‐50488H, 6′‐GNTI markedly increased the threshold for pentylenetetrazole‐induced seizures. All treated mice displayed reduced paroxysmal activity in response to U‐50488H (20 mg·kg^−1^) or 6′‐GNTI (10–30 nmoles) treatment in the mouse model of intra‐hippocampal injection of kainic acid. Single cell recordings on hippocampal pyramidal cells revealed enhanced inhibitory signalling as potential mechanisms causing the reduction of paroxysmal activity. Effects of 6′‐GNTI were blocked in both seizure models by the κ receptor antagonist 5′‐GNTI. Moreover, 6′‐GNTI did not induce CPA, a measure of aversive effects, while U‐50488H did.

**Conclusions and Implications:**

Our data provide the proof of principle that anticonvulsant/antiseizure and aversive effects of κ receptor activation can be pharmacologically separated *in vivo*.

**Abbreviations**
6′‐GNTI: 6′‐guanidinyl‐17‐(cyclopropylmethyl)‐6,7‐dehydro‐4,5α‐epoxy‐3,14‐dihydroxy‐6,7‐2′,3′‐indolomorphinan dihydrochloride
CPA: conditioned place avoidance
EKC: ethylketocyclazocine
mTLE: mesial temporal lobe epilepsy
PTZ: pentylenetetrazole
RMP: resting membrane potential
TLE: temporal lobe epilepsy

## Tables of Links

**Table:** 

**TARGETS**
**GPCRs**
κ opioid receptor

**LIGANDS**	
5′‐GNTI	EKC, ethylketocyclazocine
Dynorphin	U‐50488H

These Tables list key protein targets and ligands in this article which are hyperlinked to corresponding entries in http://www.guidetopharmacology.org, the common portal for data from the IUPHAR/BPS Guide to PHARMACOLOGY (Pawson *et al.*, 2014) and are permanently archived in the Concise Guide to PHARMACOLOGY 2015/16 (Alexander *et al.*, 2015).

## Introduction

With a prevalence of 1–2%, epilepsies belong to the most frequent neurological diseases worldwide (McNamara, 1999). Mesial temporal lobe epilepsy (mTLE) is the most frequent type of epilepsy in humans and is frequently induced by traumatic brain injury. Hippocampal sclerosis and accompanying neurological deficits are key features of mTLE (see Engel, 2001). Despite the introduction of a plethora of anti‐epileptic drugs over the last few decades, the rate of drug‐resistant focal seizures (30% and 70%) did not improve since the study of Coatsworth in 1971 (Coatsworth, 1971; Loscher and Schmidt, 2011). To date, surgical resection of the epileptogenic focus remains as the ultimate option for some of these patients. Even then, in certain subgroups of patients, less than 50% remain seizure free for at least 1 year after removal of the epileptic focus (Spencer and Huh, 2008). Thus, novel targets are needed to develop antiepileptic drugs.

Since the early 1980s, there has been evidence that some endogenous opioids, namely, dynorphin, act as modulators of neuronal excitability *in vitro* (Henriksen *et al.*, 1982; Siggins *et al.*, 1986). Because of the distribution of dynorphin in the brain, its most probable involvement in epilepsy was postulated in partial complex seizures originating from the limbic system (see Simonato and Romualdi, 1996). In line with this, the deletion of the prodynorphin coding sequence in mice (Loacker *et al.*, 2007) and low dynorphin levels in humans due to mutations in the promoter region (Stögmann *et al.*, 2002; Gambardella *et al.*, 2003) are associated with increased vulnerability to the development of epilepsy. In most animal models of temporal lobe epilepsy (TLE; comprising epilepsies arising cortical = lateral TLE and mTLE), cortical and hippocampal prodynorphin expression is reduced after an initial, short peak of over‐expression (see Simonato and Romualdi, 1996; Schwarzer, 2009). This is in line with significantly increased prodynorphin mRNA in hippocampal granule cells of patients displaying seizures within the last 48 h before surgical removal of the hippocampus, compared with those with a longer seizure‐free period (Pirker *et al.*, 2009) accompanied by an overall reduction of dynorphin immunoreactivity in surgically removed tissue obtained from mTLE patients (de Lanerolle *et al.*, 1997).

Because of the reduction of endogenous dynorphin under epileptic conditions, the κ opioid receptors may be available to bind exogenously applied agonists and the application of κ receptor agonists can suppress experimental seizures (Tortella, 1988; Takahashi *et al.*, 1990; Solbrig *et al.*, 2006; Loacker *et al.*, 2007). Various selective κ receptor agonists applied through different routes yielded time‐dependent and dose‐dependent effects similar to those upon treatment with phenytoin or phenobarbital in models of epilepsy (see Simonato and Romualdi, 1996). We recently demonstrated that the activation of κ receptors promotes the survival of hippocampal and amygdala neurons subsequent to the acute phase after unilateral injection of kainic acid in mice (Schunk *et al.*, 2011).

Along with the promising features of κ receptor activation in epilepsy models, there exists one major drawback. The therapeutic usefulness of full κ receptor agonists is decreased by their dysphoric side effects (Barber and Gottschlich, 1997). Since the failure of clinical trials with centrally active κ receptor agonists, such as spiradoline or enadoline in the 1990s, pharmaceutical companies stepped back from further development of this field. In recent years, it was suggested from animal experiments that the recruitment of β‐arrestin and subsequent phosphorylation of p38 MAPK might be crucial for the aversive effects of κ receptor activation (Bruchas *et al.*, 2007; Bruchas *et al.*, 2011). In contrast, aversive effects of κ receptor stimulation were also observed in β‐arrestin 2 knockout mice (White *et al.*, 2015), suggesting parallel pathways. In parallel, biased κ receptor agonists, which preferentially activate either the G‐protein or the β‐arrestin pathway, were reported from *in vitro* experiments. A prototypic representative of this type of compound is 6′‐guanidinyl‐17‐(cyclopropylmethyl)‐6,7‐dehydro‐4,5α‐epoxy‐3,14‐dihydroxy‐6,7‐2′,3′‐indolomorphinan dihydrochloride (6′‐GNTI), which was originally reported as a partial κ receptor agonist (Sharma *et al.*, 2001). 6′‐GNTI preferentially activates the G‐protein pathway over the β‐arrestin pathway, compared with the full agonists U‐50488H and ethylketocyclazocine (EKC) (Rives *et al.*, 2012) or U‐69,593 (Schmid *et al.*, 2013). In fact, 6′‐GNTI acts as a partial agonist for the G‐protein activation through κ receptor at low μM concentrations, but does not activate β‐arrestin in the sub‐mM range (Rives *et al.*, 2012). In contrast, RB 64 is a full agonist for G‐protein and β‐arrestin activation, although the potency for β‐arrestin is low (White *et al.*, 2015). Such drugs may open a completely new avenue in the treatment of epilepsy, provided that G‐protein biased κ receptor agonists display anticonvulsant/antiseizure properties with less aversive side effects in the complex *in vivo* situation.

The aim of this study was to investigate, whether it is possible to separate the anticonvulsant/antiseizure effects from the aversive effects by biased drugs suhc as 6′‐GNTI. For this, we applied *in vivo* EEG, electrophysiology and behavioural testing. Further, we aimed to compare the aversive effects induced by a full κ receptor agonist and by 6′‐GNTI.

## Methods

### Animals

All animal care and experimental procedures were approved by the Austrian Animal Experimentation Ethics Board in compliance with the European Convention for the Protection of Vertebrate Animals Used for Experimental and Other Scientific Purposes ETS no.: 123 and the Canadian Council on Animal Care. Every effort was taken to minimize the number of animals used. The study was designed and is reported in compliance with the ARRIVE guidelines (Kilkenny *et al.*, 2010; McGrath and Lilley, 2015).

A total of 61 C57BL/6 N wild‐type and 40 prodynorphin knockout (pDyn‐KO) mice were investigated in this study. pDyn‐KO mice were backcrossed onto the C57BL/6 N background over 10 generations (Loacker *et al.*, 2007). For breeding and maintenance, mice were group housed (maximum of five animals per cage) with free access to food and water. Temperature was fixed at 23°C and 60% humidity with a 12 h light–dark cycle (lights on 7 am to 7 pm). Male mice of the same strand and age were arbitrarily sorted into groups, splitting litters into different groups. For the animal experiments, the experimenter was blinded to the treatments of the animals.

### Pentylenetetrazole‐induced seizures

Threshold for pentylenetetrazole induced seizures was investigated in four C57BL/6 N wild‐type and 40 pDyn‐KO young adult (about 12–14 weeks; 25 g) male mice. the pentylenetetrazole threshold was measured through infusion of pentylenetetrazole (10 mg·mL in saline) through the tail‐vein in freely moving animals 20 min after intracisternal injection of either saline or different doses of 6′‐GNTI in a fixed volume of 3 μL under light sevofluorane anaesthesia. 5′‐GNTI treatment (also in 3 μL, under light sevofluorane anaesthesia) was performed 10 min prior to 6′‐GNTI in some experiments. Intracisternally saline infused animals, and animals treated with 5′‐GNTI alone, were included as control groups. Infusion was stopped at the first appearance of tonic–clonic seizures and animals were immediately killed by cervical displacement. Threshold was calculated from volume injected and body weight (Loacker *et al.*, 2007). We investigated the effects of 6′‐GNTI in this model of acute seizures because our previous finding showed that the classic κ receptor full agonist U‐50488H was able to restore the reduced pentylenetetrazole‐seizure threshold in pDyn‐KO mice.

### Kainic acid injection and electrode implantation

Twenty‐four C57BL/6 N wild‐type young adult (about 12–14 weeks; 25 g) male mice were sedated with ketamine (160 mg·kg^−1^, i.p.; Graeub Veterinary Products, Switzerland) and then deeply anaesthetized with sevoflurane through a precise vaporizer (Midmark, USA). Mice were injected with 50 nL of a 20 mM kainic acid solution into the left hippocampus as previously described (Loacker *et al.*, 2007). Four electrodes (two cortical and two depth electrodes) were implanted immediately after kainic acid administration. Epoxylite‐coated tungsten depth electrodes (diameter 250 μm; FHC, USA) were placed bilaterally into the hippocampus aimed at the CA1 area (RC −1.80 mm; ML ±1.80 mm; DV −1.60 mm). Surface electrodes were gold‐plated screws placed bilaterally into the skull on top of the motor‐cortex (RC +1.70 mm; ML ±1.6 mm with the bregma as a reference point) to monitor the generalization of abnormal EEG activities. An additional surface electrode was placed on the cerebellum as ground and reference. Electrodes were secured in place with dental acrylate cement (Heraeus Kulzer GmbH, Germany).

For experiments requiring an i.c.v. drug administration, a guide cannula was implanted in the lateral ventricle of the contralateral hemisphere (RC −0.50 mm; ML +1.00 mm; DV +2.00 mm) instead of the contralateral electrode. All animals received meloxicam (2 mg·kg^−1^) 20 min before and after surgery as an analgesic treatment. The locally injected kainic acid model was chosen because it is a model of trauma‐induced epilepsy that allows the investigation of self‐sustained paroxysmal events, and it was characterized as a model for drug‐resistant epilepsy (Riban *et al.*, 2002).

### EEG recording and analysis

The EEG was obtained using a wireless recording device (Neurologger, TSE Systems GmbH, Germany) and automatically analysed using sciworks Software (Datawave Technologies, USA). EEGs were filtered for epileptiform spikes defined as high amplitude discharges (>3 × baseline) lasting less than 70 ms. This definition allows the exclusion of other EEG events induced by hippocampal kainic acid administration, such as high‐voltage sharp waves that last considerably longer (150–200 ms; Riban *et al.*, 2002). Spike trains were defined as the occurrence of at least three spikes with a frequency higher than 1 Hz and lasting for at least 1 s. Prolonged hippocampal paroxysmal discharges (hpds) were defined as spike trains lasting for a minimum of 10 s. Spikes detected in between spike trains and hpds were counted as inter‐ictal spikes. For *in vivo* EEG, only animals displaying a minimal seizure activity of 100 s during the baseline recording were included. Therefore, group sizes may vary. EEG data are depicted as ratio of events recorded the first hour after treatment compared with the last hour before treatment. For illustration, please see the Supporting Information Fig. S1. Data regarding the U‐50488H treatment in kainic acid model obtained with the datawave software were confirmed with visual analysis.

### Conditioned place avoidance

Twenty‐eight C57BL/6 N wild‐type young adult (about 12–14 weeks; 25 g) male mice were used for the conditioned place avoidance (CPA) paradigm. Tests were conducted in a custom‐made, three‐chamber apparatus (Kummer *et al.*, 2011). The conditioning procedure comprised a pre‐test session, four consecutive training days (two training sessions per day, one each for drug or saline) and a CPA test on day six. Pre‐test and CPA test session lengths were 15 min, and the conditioning sessions lasted 30 min. If the pre‐test presented a bias for one of the two chambers, the preferred one was paired with the drug (6′‐GNTI or U‐50488H) to facilitate the detectability of avoidance.

Prior to the conditioning sessions, the animals were treated with one of the drugs tested or saline in a neutral chamber. All experiments were performed with an illumination of 60 Lux in the centre of the conditioning chambers. The time spent in each compartment was analysed with the VideoMot 2 System (TSE Systems GmbH). To analyse the influence of drug treatment on motor activity, the distance travelled in the drug‐paired or saline‐paired chamber was recorded on the first treatment day using the VideoMot 2 System.

### Immunohistochemistry

Immunohistochemistry was performed as described elsewhere (Schwarzer *et al.*, 1995). In brief, mice were killed 30 min after drug treatment (*n* = 4 per group) by an overdose of thiopental (150 mg·kg^−1^), and brains were fixed by transcardial perfusion with 4% paraformaldehyde in PBS (50 mM PBS, pH 7.2). Immunohistochemistry was performed on coronal 40 μm vibratome sections incubated free‐floating in blocking solution (10% normal goat serum and 0.3% Triton X‐100 in TBS) for 90 min. Primary antibodies against Zif268 (1:2000; Santa Cruz; # sc189) or pERK (1:400; Cell Signalling Technology; #9101) were applied overnight at room temperature followed by horseradish peroxidase conjugated secondary antibodies (1:500, Dako) and 3,3′‐diaminobenzidine for detection. Specificity of antibodies was controlled by avoiding primary antibodies or by pre‐absorbing the primary antibody with the respective blocking peptide (Zif 268, Santa Cruz, # sc189 P; pERK 1, Cell Signalling, #9101; 10 μg mL) overnight at +4°C before the application on the section.

### Electrophysiology

Five C57BL/6 N male wild‐type mice (25 g) between 8–16 weeks of age were used for *in vitro* electrophysiology. Mice were decapitated; their brains rapidly removed and submerged in an icy slurry of artificial CSF (ACSF) optimized for slice preparation (slice solution), containing (in mM) 118 NaCl, 3 KCl, 1.3 MgSO_4_, 1.4 NaH_2_PO_4_, 10 glucose, 26 NaHCO_3_ and 2.5 CaCl_2_, which was bubbled continuously with carbogen (95% O_2_, 5% CO_2_). Kynurenic acid (1 mM) was also added to the slice solution to reduce glutamate‐mediated excitotoxicity. Mid‐ventral, transverse sections of the hippocampus (300 μm) were cut using a vibrating slicer (Slicer HR2; Sigmann Elektronik). Slices were then transferred to a carbogenated ACSF (bath) solution, which contained the following (in mM): 124 NaCl, 3 KCl, 1.3 MgSO_4_, 1.4 NaH_2_PO_4_, 10 glucose, 26 NaHCO_3_ and 2.5 CaCl_2_ (300–305 mOsm·L^−1^). Slices were stored at room temperature (22°C) in the bath solution for at least 30 min following slicing; the bath solution was also used to perfuse slices for all experiments. Slices were placed into a recording chamber attached to a fixed stage of a moveable upright microscope (Axioskop FS2; Carl Zeiss, Vienna, Austria) and held submerged by a platinum and polyester fibre ‘harp’. Slices were continuously perfused with a warmed (34 ± 0.5°C), carbogenated bath ACSF at a rate of 2–3 mL·min^−1^ for at least 20 min prior to recording.

Pipettes were pulled from thin‐walled borosilicate glass (TW150F; WPI, Sarasota, FL, USA) with a two‐stage puller (PP‐83; Narishige, Amityville, NY) to a tip resistance of 5–7 MΩ when backfilled with an internal solution containing (in mM) 126 K‐gluconate, 10 HEPES, 4 KCl, 5 MgATP, 0.3 NaGTP, 1 EGTA and 0.3 CaCl_2,_ to which 0.05–0.1% neurobiotin was added, and the pH was adjusted to 7.27–7.30 with KOH, (275–285 mOsm·L^−1^). Recordings were made using a Multiclamp 700B amplifier data and were acquired using a pCLAMP 10.3 via a Digidata 1322 interface (Molecular Devices, Sunnyvale, CA).

CA3 and CA1 pyramidal neurons were visually identified using infrared differential interference contrast optics and selected for recordings based on characteristic pyramidal morphology and the presence of a large apical dendrite. After obtaining the whole cell patch clamp configuration, neurons were held in a voltage clamp at −60 mV for 5–10 min before beginning experiments and between experimental measurements. Only neurons that showed stable resting membrane potential (RMP), holding a current (in a voltage clamp) in a series of control measurements, and which showed stable access resistance throughout the entire experiment, were selected for analysis.

A stimulation isolation unit (Iso‐Flex, AMPI, Jerusalem) was used to elicit synaptic responses onto recorded neurons. Stimulating electrodes (bath ACSF‐filled patch pipettes) were placed in either the mossy fibre layer for CA3 recordings or the Shaffer collaterals for CA1 recordings, exciting axons from the dentate gyrus and CA3 respectively. Stimulation intensities were adjusted to evoke responses of approximately half‐maximal amplitude. Synaptic responses that were evoked as neurons were held at −40 mV, which allowed inhibitory (likely GABAergic) and excitatory responses to be differentiated as outward and inward currents respectively. At −40 mV, most neurons showed both evoked EPSCs and inhibitory postsynaptic current (IPSCs). Holding neurons at −60 mV eliminated the IPSC component of evoked responses, suggesting the IPSC was largely GABA_A_ receptor‐mediated. Spontaneous IPSCs and EPSCs were also measured during 2 min continuous voltage clamp recordings at −40 mV. The RMP of neurons was measured by averaging the potential over a 30 s period of passive current clamp recording. Drug effects on postsynaptic conductances were measured using a series of hyperpolarizing voltage steps conducted from a holding potential of −40 mV. Eight successive hyperpolarizing steps were used; the initial step hyperpolarized the membrane 10 mV, and each successive step increased by a −10 mV increment such that the final step moved the membrane from −40 to −120 mV. Each successive hyperpolarizing step was shortened by Xms to limit voltage‐mediated damage to the cell membrane. Several (>2) sets of recordings of neuronal and synaptic properties (evoked synaptic responses, etc.) were taken at 5 min intervals before drug application.

6′‐GNTI, dissolved in 10 mL ACSF to a final concentration of 1 μM, was applied via bath perfusion over a period of 2–3 min and then was washed out with ACSF. Recordings were taken immediately prior to drug application, during application, 1 min after application and successively every 5 min until drug effects washed out. The electrophysiology was performed unblinded due to practical reasons.

### Data and statistical analysis

The data and statistical analysis comply with the recommendations on experimental design and analysis in pharmacology (Curtis *et al.*, 2015). Data are presented as mean ± SEM. Following acquisition, electrophysiological recordings were viewed and analysed using pClamp 10.3 (Molecular Devices). Prism 5 for Mac (version 5.0f) was used to perform a statistical analysis of *in vivo* experiments and to generate figures. For the statistical analysis, a one‐way ANOVA with a Dunnett *post hoc* test was applied to *in vivo* experiments. For electrophysiology, the two‐tailed, paired *t*‐tests were applied for RMP analysis, and a one‐way ANOVA was used to compare drug effects on IPSC. CPA data were analysed using paired *t*‐test and two‐way ANOVA with a Bonferroni *post hoc* test. A *P* value lower than 0.05 was considered significant.

### Materials

Diazepam and pentylenetetrazole were purchased from Sigma Aldrich (St. Louis, MO). The κ receptor agonist U‐50488H and antagonist 5′‐GNTI were purchased from Tocris Bioscience, (Bristol, UK) while the G‐protein biased partial κ receptor agonist 6′‐GNTI was provided by the drug supply programme of National Institute on Drug Abuse (NIDA). pentylenetetrazole, U‐50488H, 5′‐GNTI and 6′‐GNTI were dissolved in saline, and the pH was adjusted to 7.2. Diazepam was dissolved in DMSO (10 mg·mL) and further diluted with saline. Diazepam and U‐50488H were applied i.p. For EEG recordings and CPA experiments, 5′‐GNTI (3 or 10 nmoles per 3 μL) and 6′‐GNTI (1–30 nmoles per 3 μL) were given i.c.v., while for the pentylenetetrazole experiments, they were administered intracisternally.

## Results

**Figure 1:**
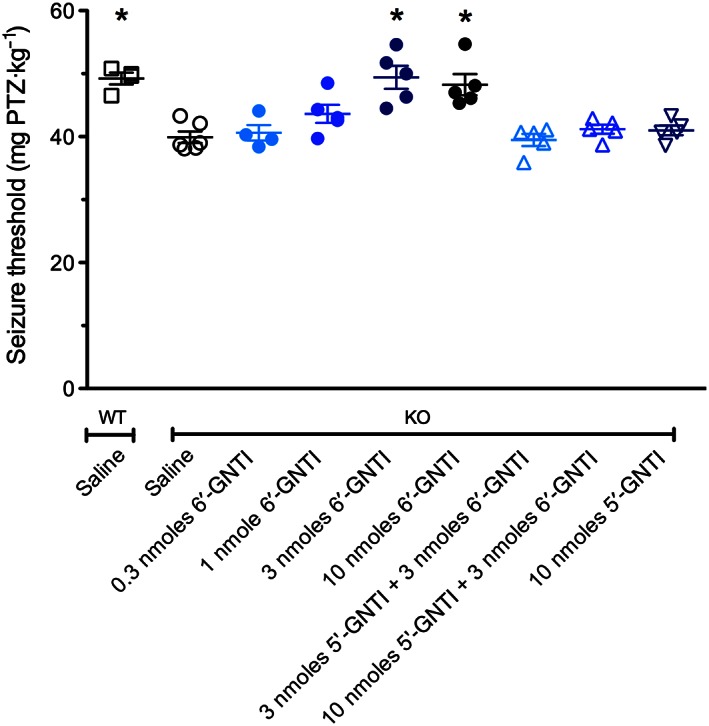
The dose–response curve of 6′‐GNTI on the threshold of pentylenetetrazole‐induced seizures as obtained by tail‐vein infusion. Twenty minutes after i.c.v. injection of 3 (*n* = 5) or 10 nmoles (*n* = 5) 6′‐GNTI, the seizure threshold was increased, compared with saline‐treated pDyn‐KO (*n* = 6) animals. No effect was observed at lower doses (0.3 nmole *n* = 4; 1 nmole *n* = 5). The seizure threshold of pDyn‐KO mice treated with 3 or 10 nmoles 6′‐GNTI was undistinguishable from that of saline‐treated wild‐type mice (*n* = 4). Pretreatment of pDyn‐KO animals with 5′‐GNTI (3 nmoles *n* = 5; 10 nmoles *n* = 5) 10 min before 6′‐GNTI entirely reversed this effect, while 5′‐GNTI alone (*n* = 5) had no effect on the seizure threshold. *P < 0.05, significantly different from saline; one‐way ANOVA with Dunnett's *post hoc* test. WT, wild type; KO, prodynorphin knockout.

Based on our previous finding that the full κ receptor agonist U‐50488H was able to restore the reduced pentylenetetrazole‐seizure threshold in pDyn‐KO mice, we investigated the effects of 6′‐GNTI in this model of acute seizures. As shown in Figure 1, 6′‐GNTI dose‐dependently increased the seizure threshold in pDyn‐KO mice (one‐way ANOVA, *F*_8,43_ = 11.37). This effect reached a plateau at about 3 nmoles (*P* < 0.05). Pretreatment of animals with 3 or 10 nmoles of the specific κ receptor antagonist 5′‐GNTI reversed this effect.

**Figure 2:**
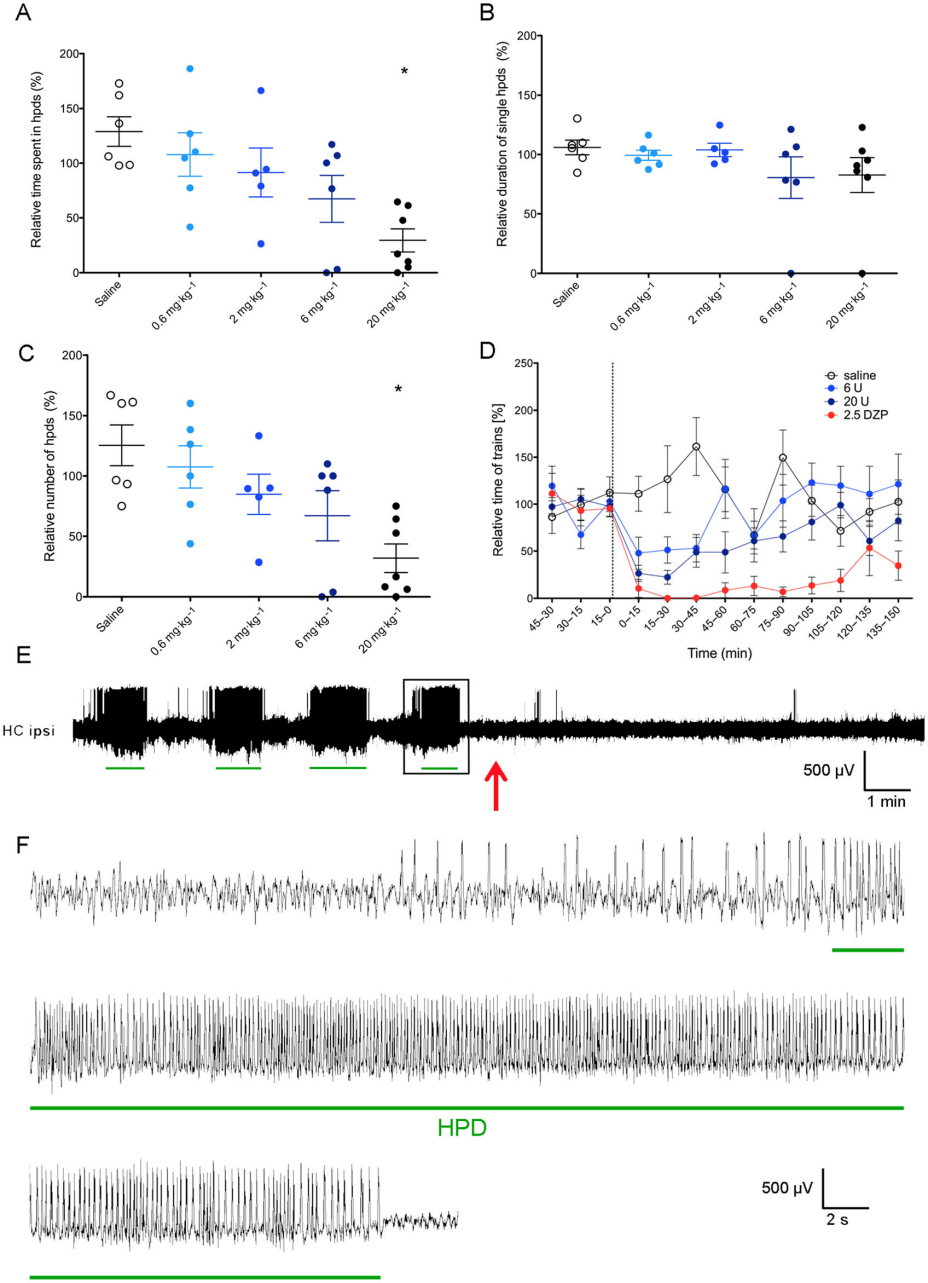
Effects of treatment with U‐50488H on time (A), average duration (B) and number (C) of hpds in kainic acid‐injected mice (*n* = 10). Ratios of total recorded events 60 min post‐treatment/60 min pretreatment are given. (D) Time course of spike trains after saline, U‐50488H (6 and 20 mg·kg^−1^) and diazepam treatments. Data are presented as ratio of total time of spike trains at time interval indicated/average time of paroxysmal discharges of the three 15 min intervals before treatment. The time of treatment is indicated by the dotted line. (E) Representative EEG trace showing spike trains recorded from the ipsilateral hippocampus in the 15 min periods preceding and following a 20 mg·kg^−1^ U‐50488H treatment. Time of U‐50488H administration is indicated with a red arrow. Green lines indicate the EEG parts detected as hpds by the analysis software. (F) Higher magnification of the hpds framed in panel E. *P < 0.05, significantly different from saline; one‐way ANOVA with Dunnett's *post hoc* test. DZP, diazepam; U, U‐50488H; HC ipsi, ipsilateral hippocampus.

Unilateral injection of kainic acid into the dorsal hippocampus of mice was characterized as a model for drug‐resistant epilepsy based on the non‐responsiveness of paroxysmal discharges to acute carbamazepine, phenytoin or valproate treatment (Riban *et al.*, 2002). This, at least for some individuals, holds true also for modern antiepileptic drugs (Supporting Information Fig. S2). In our study, animals injected with 1 nmole of kainic acid developed hpds within 3–4 weeks after treatment. Generalized seizures were observed rarely (1–3 seizures per day per animal), while hpds occurred 23 ± 3.6 times per hour and lasted for 17.5 ± 1.4 s (*n* = 21). During hpds, mice displayed behavioural arrest and stereotypies. To investigate the potentials of κ receptor agonists in this model, we first tested whether the full κ receptor agonist U‐50488H affected these EEG abnormalities. As shown in Figure 2A–C, treatment with U‐50488H decreased hpds. A reduction in the number of hpds (one‐way ANOVA, *P* < 0.05 for 20 mg·kg^−1^; *F*_4,29_ = 5.081) resulted in a significant reduction of total time spent in hpds (one‐way ANOVA, *P* < 0.05 for 20 mg·kg^−1^; *F*_4,29_ = 5.221). In contrast to diazepam‐treated mice, which were not moving (most probably due to the highly sedative effects of diazepam), U‐50488H‐treated animals were actively exploring the cage during this time. The onset of suppression of spike trains is very rapid for both diazepam and U‐50488H, as shown in Figure 2D.

U‐50488H, as a prototypic specific full κ receptor agonist, was appropriate to assess the potential of κ receptors in the kainic acid model. However, U‐50488H is known to produce aversive effects, which need to be overcome to consider the application of κ receptor agonists in humans. These aversive effects were reported to depend on the recruitment of the β‐arrestin and subsequent phosphorylation of p38α‐MAPK (Bruchas *et al.*, 2007; Land *et al.*, 2008) in mice. To examine whether anticonvulsive and antiseizure effects can be elicited through κ receptor activation, independent of the activation of the β‐arrestin pathway, we investigated the G‐protein biased partial κ receptor agonist 6′‐GNTI, which activates G‐protein mediated effects of κ receptor with low recruitment of β‐arrestin (Rives *et al.*, 2012).

**Figure 3:**
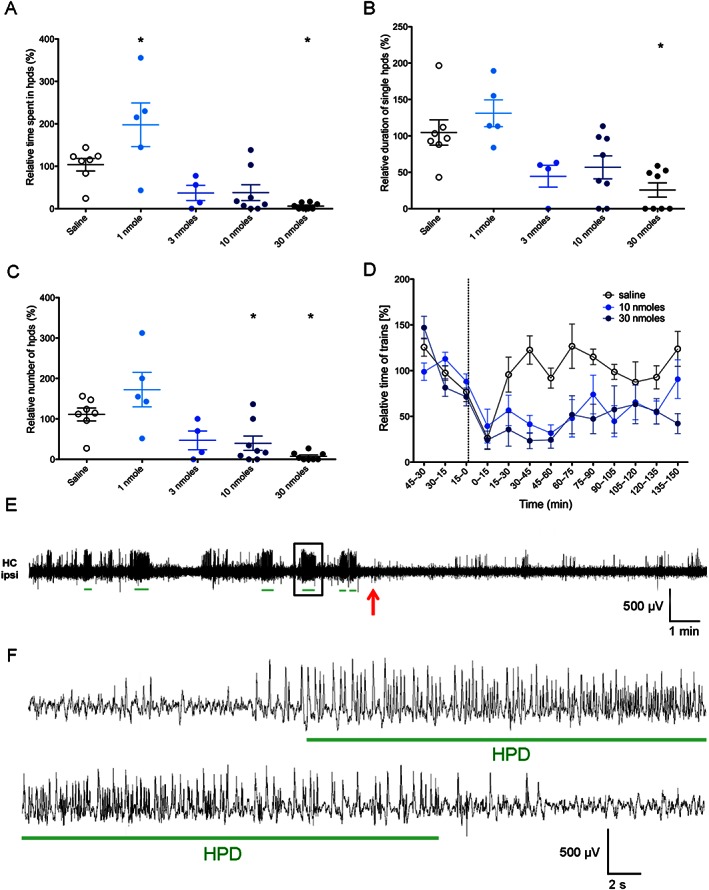
Effects of 6′‐GNTI treatments on time (A), average duration (B) and number (C) of hpds in kainic acid‐injected mice (*n* = 14). Ratios of total recorded events 60 min post‐treatment/60 min pretreatment are given. (D) Time course of spike trains after saline and 6′‐GNTI (10 and 30 nmoles) treatments. Data are presented as ratio of total time of EEG abnormalities at time interval indicated/average time of paroxysmal discharges of the three 15 min intervals before treatment. The time treatment is indicated by the dotted line. (E) Representative EEG trace showing spike trains recorded from the ipsilateral hippocampus in the 15 min periods preceding and following a 30 nmoles 6′‐GNTI treatment. Time of 6′‐GNTI administration is indicated with a red arrow. Green lines indicate the EEG parts detected as hpds by the analysis software. (F) Higher magnification of the hpds framed in panel E. *P < 0.05, significantly different from saline; one‐way ANOVA with Dunnett's *post hoc* test. HC ipsi, ipsilateral hippocampus.

As shown in Figure 3A–C, 6′‐GNTI reduced hpds, affecting both duration and number of events. The event duration (one‐way ANOVA, *P* < 0.05 for 30 nmoles; *F*_4,31_ = 7.582) as well as the number of events (one‐way ANOVA, *P* < 0.05 for 10 nmoles and *P* < 0.05 for 30 nmoles; *F*_4,31_ = 9.854) were reduced, resulting in a significant reduction of total time of hpds (one‐way ANOVA, *P* < 0.05 for 30 nmoles; *F*_4,31_ = 10.59). Although 6′‐GNTI is only a partial κ receptor agonist, animals displayed a strong reduction of spike trains after the treatment with 10 or 30 nmoles (Figure 3D). This effect was fully blocked by the κ receptor antagonist 5′‐GNTI (Supporting Information Fig. S4). Comparable results were obtained when EEGs were analysed for inter‐ictal spikes (Table 1).

**Table 1:** Effects of U‐50488H and 6′‐GNTI on inter‐ictal spikes and spike trains. *P <0.05, significantly different from saline.

		Inter‐ictal spikes		
Saline	0.6 mg·kg^−1^ U‐50488H	2 mg·kg^−1^ U‐50488H	6 mg·kg^−1^ U‐50488H	20 mg·kg^−1^ U‐50488H
98.03 ± 8.69% (*n* = 6)	106.4 ± 10.1% (*n* = 7)	82.57 ± 14.78% (*n* = 6)	92.92 ± 11.04% (*n* = 7)	103.3 ± 9.95% (*n* = 7)
Saline	1 nmole 6′‐GNTI	3 nmole 6′‐GNTI	10 nmole 6′‐GNTI	30 nmole 6′‐GNTI
162.3 ± 37.19% (*n* = 8)	114.5 ± 28.3% (*n* = 6)	76.94 ± 8.79% (*n* = 8)	137.7 ± 24.35% (*n* = 11)	67.88 ± 8.29% (*n* = 9)*
		**Spike trains**		
Saline	0.6 mg·kg^−1^ U‐50488H	2 mg·kg^−1^ U‐50488H	6 mg·kg^−1^ U‐50488H	20 mg·kg^−1^ U‐50488H
126.2 ± 4.49% (*n* = 6)	111.6 ± 11.08% (*n* = 8)	95.85 ± 9.796% (*n* = 7)	72.35 ± 18.63% (*n* = 7)*	38.68 ± 10.34% (*n* = 8)*
Saline	1 nmole 6′‐GNTI	3 nmole 6′‐GNTI	10 nmole 6′‐GNTI	30 nmole 6′‐GNTI
105.6 ± 4.606% (*n* = 8)	123.8 ± 14.31% (*n* = 7)*	75.11 ± 12.94% (*n* = 8)	62.04 ± 15.38% (*n* = 10)*	27.68 ± 10.05% (*n* = 8)*

**Figure 4:**
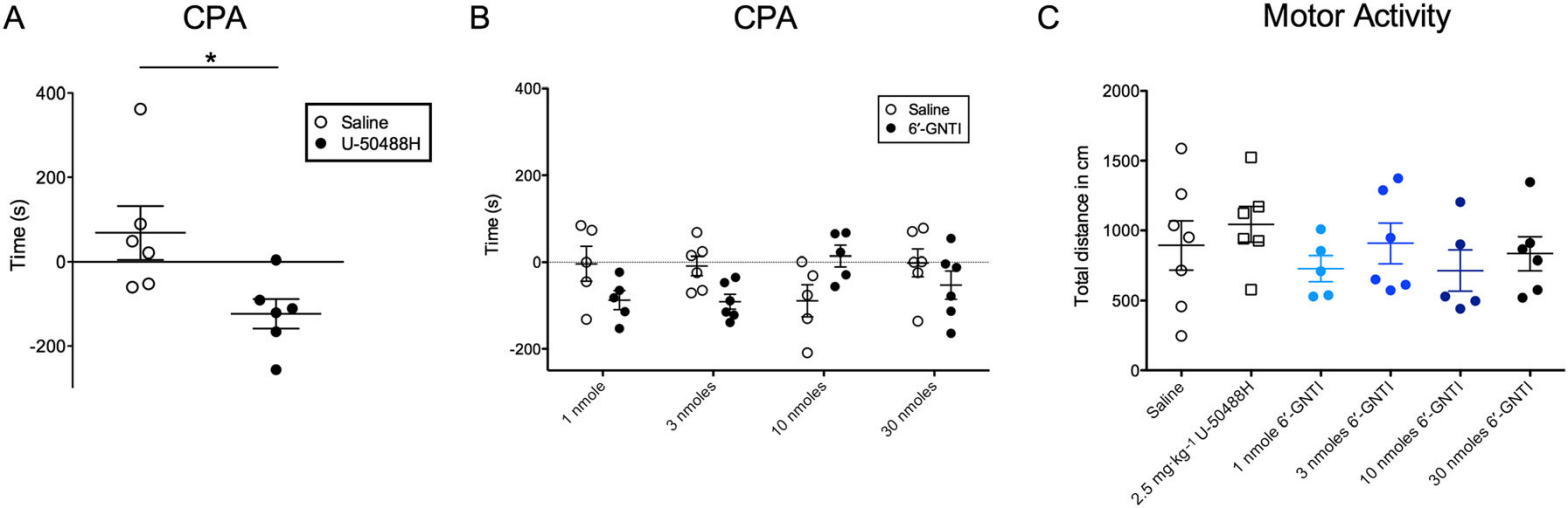
CPA for U‐50488H (A; *n* = 6) and different doses of 6′‐GNTI (B; each dose *n* = 5–6). Time is calculated as time spent in the drug‐paired compartment post test minus pre‐test.. *P < 0.05, significantly different from saline; two‐way ANOVA with Bonferroni's *post hoc* test. (C) Analysis of motor activity in the first 10 min following administration of saline, U‐50488H and different doses of 6′‐GNTI. None of the treatments revealed a statistical significant effect.

Activation of κ receptors with the full agonist U‐50488H induces strong place avoidance. No such data were available for 6′‐GNTI. Therefore, we applied a CPA paradigm to investigate potential aversive effects of 6′‐GNTI treatment. As expected, U‐50488H (2.5 mg·kg^−1^) induced strong place avoidance (Figure 4A; saline 68 ± 63.3 s; U‐50488H −123 ± 35.0 s; *n* = 6; Mann–Whitney *P* < 0.05). In contrast, 6′‐GNTI induced neither avoidance nor preference for the drug‐conditioned chamber at any tested dose (Figure 4B). Moreover, 6′‐GNTI did not influence motor activity of mice at the doses tested (Figure 4C).

To investigate whether lack of aversive effects might be resulting from local application and restricted spread of the drug, we investigated the expression of the protein Zif268 (known to be activated by κ receptors, 30 min after treatment) with saline, 2.5 mg·kg^−1^ U‐50488H or 30 nmoles 6′‐GNTI. Strong Zif268‐immunoreactivity was observed in various regions of the brain, most prominent in the striatum, nucleus accumbens (Supporting Information Fig. S3) and cortical regions in the 6′‐GNTI group and to a lower level in the U‐50488H group. At this time interval, phosphorylation of ERK1 was hardly detectable. Of note is the fact that several neurons in the dorsal raphe displayed pERK immunoreactivity in the 6′‐GNTI group (Supporting Information Fig. S3).

**Figure 5:**
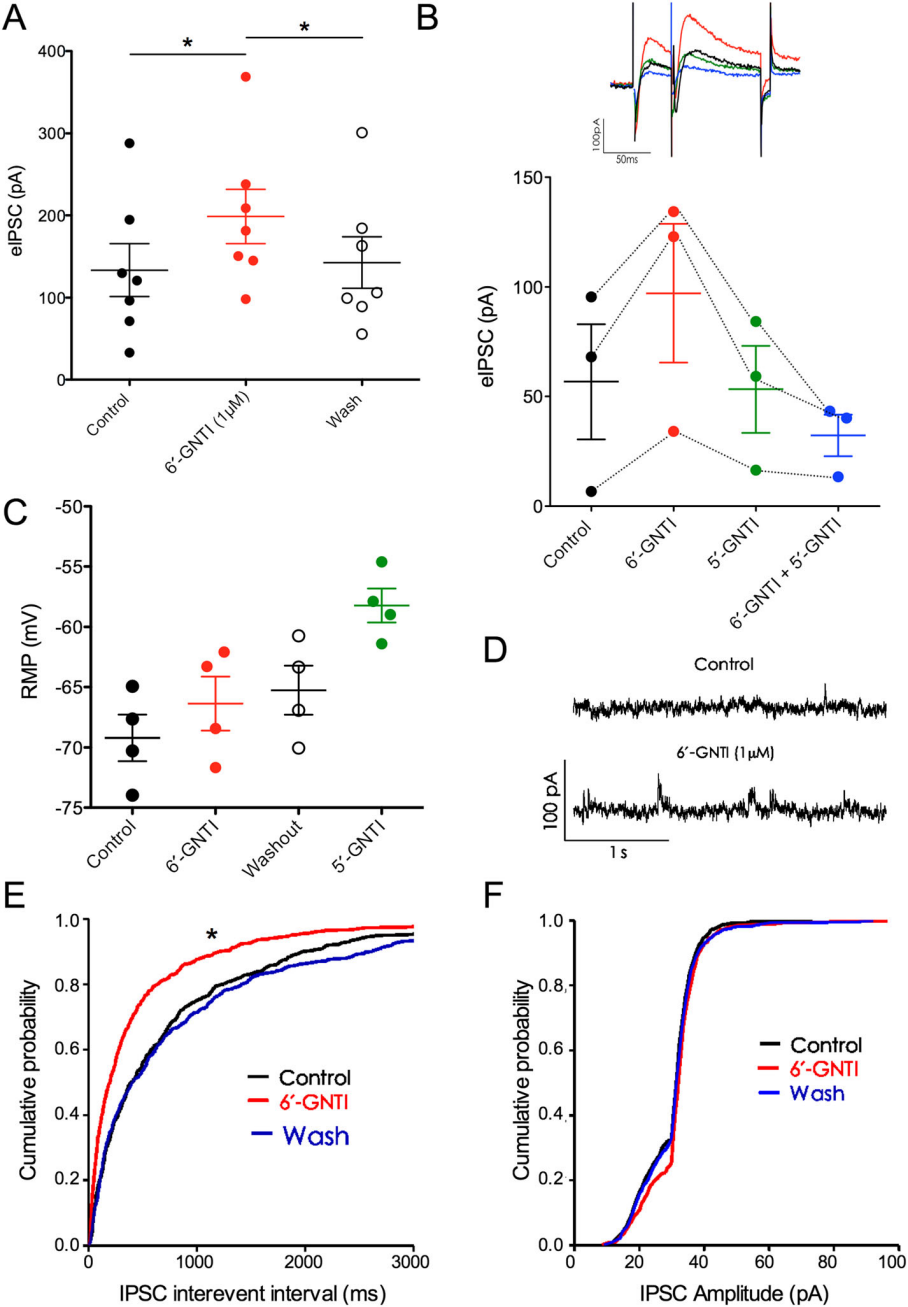
6′‐GNTI effects on hippocampal pyramidal neurons. (A) 6′‐GNTI treatment (1 μM) increased the amplitude of evoked inhibitory post‐synaptic current (eIPSC) in pyramidal cells (*n* = 7). The effect was reversed upon washout of the drug. *P < 0.05, significantly different from control; one‐way ANOVA with Dunnett's *post hoc* test. (B) Top: representative traces showing eIPSC under different conditions. Bottom: bath application of 5′‐GNTI alone and in the presence of 6′‐GNTI (*n* = 3). (C) RMP was not affected by 6′‐GNTI (*n* = 4). (D) Representative traces showing spontaneous IPSCs in control and 6′‐GNTI treated slices. (E) Increased spontaneous IPSCs frequency in pyramidal cells upon 6′‐GNTI bath perfusion (*n* = 5). (F) Spontaneous IPSCs amplitudes on pyramidal cells (*n* = 5). In (E ) and (F), *P < 0.05, significantly different from control; one‐way ANOVA with Kolmogorov–Smirnov test.

Although 6′‐GNTI was first described several years ago (Sharma *et al.*, 2001), no data on its effects on hippocampal pyramidal neurons are available. Therefore, we investigated 6′‐GNTI effects on hippocampal slices. Stimulation of hippocampal mossy fibers evoked IPSCs in CA3 pyramidal cells with an amplitude of 134 ± 32 pA. In the presence of 1 μM 6′‐GNTI, the amplitude of evoked IPSC significantly increased to 199 ± 33 pA (Figure 5A; *n* = 7; one‐way ANOVA, *P* < 0.05 for 6′‐GNTI vs. control; *F*_2,20_ = 23.28), the effect was reversed upon washout of the drug. In a separate experiment, we tested whether the specific κ receptor antagonist 5′‐GNTI was able to block the increased eIPSC amplitude. After 6′‐GNTI application (demonstrating the responsiveness of cells to 6′‐GNTI), slices were bathed in ACSF containing 5′‐GNTI but not 6′‐GNTI. Under these conditions, eIPSC amplitudes were on control level (Figure 5B). Evoked IPSC amplitude did not change upon reapplication of 6′‐GNTI in the presence of 5′‐GNTI (Figure 5B), suggesting that the κ receptor dependence of the 6′‐GNTI effect on eIPSC amplitude.

In addition, 6′‐GNTI increased the frequency of spontaneous IPSCs in pyramidal cells. Mean frequency was significantly increased by 6′‐GNTI from 1.2 ± 0.1 to 2.1 ± 0.2 Hz (Figure 5E; *n* = 5; *P* < 0.05; K‐S test), and reversed on washout. No effect on pyramidal cell spontaneous IPSCs amplitudes was observed (Figure 5F).

In this series of neurons, 6′‐GNTI did not affect the RMP. However, 5′‐GNTI shifted the RMP in 4/5 pyramidal neurons tested from −70.5 ± 1.3 to −58.2 ± 1.4 mV (Figure 5C).

## Discussion

Treatment of mice injected with kainic acid with the κ receptor agonist U‐50488H results in reduced neuronal loss (Schunk *et al.*, 2011) and reduces hippocampal paroxysmal discharges (this study). However, general κ receptor agonists induce dysphoria in humans (Barber and Gottschlich, 1997) and aversive effects in mice (Land *et al.*, 2008). These aversive effects were suggested to be dependent on the β‐arrestin binding to κ receptors and subsequent activation of p38 MAPK in mice (Bruchas *et al.*, 2007; Carr and Mague, 2008). In contrast, the full κ receptor agonists U69593 and salvinorin A as well as the G‐protein biased agonist RB‐64 (a salvinorin A derivative) produced pronounced aversive effects also in β‐arrestin 2 knockout mice (White *et al.*, 2015). Activation of p38 MAPK was not addressed in this study, thereby not answering the question whether p38 MAPK could have been activated through other mechanisms (i.e. β‐arrestin 1). RB‐64 is a full agonist for G‐protein and β‐arrestin activation, although the potency for β‐arrestin is low (White *et al.*, 2015). The G‐protein biased partial κ receptor agonist 6′‐GNTI activates the β‐arrestin pathway at an almost negligible level, resulting in a lack of ERK1/2 phosphorylation in striatal neurons (Schmid *et al.*, 2013). The remaining activation of the Akt pathway was suggested to be G‐protein dependent. Interestingly, we observed strong expression of Zif268 in the striatum and other brain regions after application of 6′‐GNTI, but we cannot provide an underlying mechanism for this effect. Regulation of Zif268 expression potentially involves activation of PKA, PKC or CaMK signalling, involving either MAPK/ERK or CREB‐dependent mechanisms (see Bozon *et al.*, 2002).

6′‐GNTI was described as a partial agonist for G‐protein activation and inhibition of adenylate cyclase, with hardly any activation of β‐arrestin compared with full agonists such as U‐50488H and EKC, in HEK293T cells transfected with human κ receptors (Rives *et al.*, 2012). In our experiments, 6′‐GNTI displayed significant elevation of seizure threshold and reduction in hpds activity without induction of CPA, a measure of aversive effects, or motor impairment *in vivo*. Marked and widespread expression of Zif268 suggests that i.c.v. application of 6′‐GNTI actually activates κ receptors in areas proposed to be responsible for dysphoric effects, thereby suggesting that lack of aversive effects is not due to the local application. In contrast, RB‐64 induced CPA (White *et al.*, 2015). As pointed out earlier, the pharmacological profiles of these two drugs differ. However, the route of administration might also influence this difference because, in our study, 6′‐GNTI was given i.c.v., while White *et al.* (2015) administered RB‐64 systemically. As CPA measures any type of unpleasant effects, we cannot exclude the possibility that activation of peripheral κ receptors by RB‐64 may cause the CPA. Moreover, it cannot be concluded that biased drugs display the same pharmacodynamic features *in vitro* and *in vivo*, as this bias may depend on the microenvironment of the receptor.

The fast onset of response suggests that activation of the G‐protein, but not the β‐arrestin pathway, is essential for anticonvulsant or antiseizure effects of κ receptor agonists. This is in line with the observations that presynaptic activation of κ receptors decreases N‐type, L‐type and P/Q‐type Ca^2^^+^ currents (Rusin *et al.*, 1997) most probably through direct interaction with the βγ subunit of the G‐protein, resulting in a reduction of glutamate release. Stimulation of voltage‐gated K^+^ channels through postsynaptic κ receptors was proposed to occur in pyramidal neurons (Moore *et al.*, 1994; Madamba *et al.*, 1999). Our electrophysiological data do not clearly support these possibilities. By contrast, increased inhibitory signalling may be responsible for seizure suppression. GABAergic interneurons express not only κ receptors (Racz and Halasy, 2002) but also δ opioid receptors (Commons and Milner, 1996). It is unclear whether δ receptor and κ receptors form heterodimers that could bind 6′‐GNTI (Waldhoer *et al.*, 2005) or interfere with excitation of interneurons. Detailed analysis of this aspect, including the question whether the effect results from the direct stimulation of GABAergic neurons or from an indirect stimulation through increased firing of excitatory inputs on GABAergic neurons, is beyond the scope of this study. 6′‐GNTI shows a high affinity for the κ receptor/δ receptor and κ receptor/μ opioid receptor heterodimers, as well as for κ receptor homodimers *in vitro* (Waldhoer *et al.*, 2005). In line with this, the blockade of κ receptors by the specific antagonist 5′‐GNTI abolished the effect of 6′‐GNTI.

Translatability is always an issue when it comes to animal models. This is especially true in the case of structural differences between the human and the animal drug target. There are differences between human and rodent κ receptors in terms of β‐arrestin recruitment (Schattauer *et al.*, 2012). Importantly, 6′‐GNTI does not induce recruitment of β‐arrestin in HEK cells expressing human κ receptors (Rives *et al.*, 2012). This is in line with the lack of CPA in this study, considering the proposed functional relationship between β‐arrestin recruitment and aversive effects (Bruchas *et al.*, 2007). As observed in rodents, *in vitro* experiments on human κ receptors expressed in CHO cells demonstrated partial agonist activity of 6′‐GNTI on the binding of [^35^S]‐GTP‐γS (Schmid *et al.*, 2013), suggesting activation of the G‐protein.

Local injection of kainic acid into the area CA1 of the dorsal mouse hippocampus was described as a drug‐resistant model of TLE (Riban *et al.*, 2002). Trauma‐induced epilepsies often comprise partial complex seizures originating from the limbic system, for which implications of the dynorphinergic system were postulated (see Simonato and Romualdi, 1996). Rodents and humans display a similar pattern of dynorphin expression and κ receptor binding in the hippocampus. Most animal models of TLE reflect the dynamics of post‐ictal overexpression and inter‐ictal reduction in dynorphin mRNA and peptide as described in human mTLE (see Simonato and Romualdi, 1996; Schwarzer, 2009). Alterations in κ receptor binding in epilepsy appear mostly dependent on neuronal loss, but not on a change in expression or trafficking. Thus, patients suffering from mass‐associated TLE or paradoxical TLE did not show marked differences in κ receptor specific [^3^H]U‐69,593 binding compared with post‐mortem controls. In contrast, hippocampi of mTLE patients displayed reduced binding in area CA1, but not the subiculum, being in line with marked neuronal loss in CA1 but not in the subiculum (de Lanerolle *et al.*, 1997). In addition, in rodents, alterations mainly reflect morphological changes due to the death of κ receptor‐expressing neurons in the hippocampus in epilepsy (see Ben‐Ari, 2001). Reduced amounts of dynorphin but unaltered levels of κ receptors might result in an increased number of receptors available for agonist treatment. Due to the well‐documented and parallel findings in mouse and man, we expect a highly predictive value of experimental data obtained from animal models for functional implications in humans.

There is no general agreement as to which parameter of animal models might have the highest predictive value for human epilepsies. Therefore, we include different settings for the EEG analysis to analyse inter‐ictal spikes and spike trains separately. Moreover, we filtered for seizure‐like events of 10 s or more (hippocampal paroxysmal discharges), as these were suggested to have higher informative values in terms of human seizures. Irrespective of the settings, 6′‐GNTI induced reduction of inter‐ictal spikes and seizure‐like events, at least at the highest dose tested. In contrast, the modern antiepileptic drugs showed significant reduction of spike trains, but were mostly ineffective on hippocampal paroxysmal discharges.

In conclusion, our data provide proof for the principle that anticonvulsant and antiseizure effects can be achieved without inducing place‐aversion by applying a partial κ receptor agonist, which preferentially activates the G‐protein over the β‐arrestin pathway, at least *in vitro*.

## Conflict of interest

The authors declare no conflicts of interest.

## Declaration of transparency and scientific rigour

This Declaration acknowledges that this paper adheres to the principles for transparent reporting and scientific rigour of preclinical research recommended by funding agencies, publishers and other organisations engaged with supporting research.

## Supporting information


**Figure S1** Treatment design for U‐50488H and 6′‐GNTI applied on kainic acidinjected mice (referring to Figures 2 and 3). Data were calculated as ratio of the 60 min following the treatment on the 60 min preceding the treatment. (B) Representation of the automatic detection of spikes, spike trains and hpds. Spikes were defined as high amplitude discharges (>3 x baseline) lasting less than 70 ms. Spike trains were defined as the occurrence of at least three spikes with a frequency higher than 1 Hz and lasting for at least 1 s. Dashed green line =0 μV; green line = baseline; red line = threshold (3 x baseline).
**Figure S2** Effects of antiepileptic drug treatments on time of hpds on kainic acid injected mice (n = 12). Ratios of total recorded events 60 min post/60 min pre‐treatment are given. * *P* < 0.05, significant difference; one‐way ANOVA with Dunnett's *post hoc* test..
**Figure S3** Immunohistochemistry for Zif268 (upper panel, depicting nucleus accumbens) and pERK1 (lower panel, depicting dorsal raphe) was performed 30 minutes after i.c.v. application of saline (a, e; n = 4), 2.5 mg/kg U‐ 50488H (b, f; n = 4) or 30 nmoles 6′‐GNTI (c, d, g, h; n = 4). Note the strong labelling for Zif268 in the nucleus accumbens and the moderate labeling for pERK1 in the dorsal raphe after 6′‐GNTI injection. In d and h the primary antibodies, Zif 268 (n = 4) and pERK 1 (n = 4) respectively, were pre‐absorbed with the blocking peptide, resulting in a lack of labeling as compared to c and g (sections obtained from the same brains). Scale bars in c for a‐d = 50 μm, in g for e‐h = 25 μm.
**Figure S4** Effects of 6′‐GNTI (30 nmoles/3 μl; n = 8) and 5′‐GNTI treatments (10 nmoles/3 μl; n = 5) on time of hpds on kainic acid injected mice compared t saline (n = 7). 6′‐GNTI effect was entirely reversed upon coadministration with 5′‐GNTI (n = 4). Instead, 5′‐GNTI alone (n = 5) had no effect on the time spent in hpds. * *P* <0.05, significant difference; one‐way ANOVA with Bonferroni's *post hoc* test.

Supporting info item

Supporting info item

Supporting info item

Supporting info item
