# Supplementary figures and images for: The G‐protein biased partial κ opioid receptor agonist 6′‐GNTI blocks hippocampal paroxysmal discharges without inducing aversion
Source: Br J Pharmacol. 2016 Apr 21;173(11):1756–67. doi: 10.1111/bph.13474 (PMC4867738; doi:10.1111/bph.13474)

A

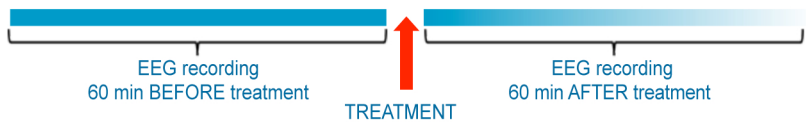

B

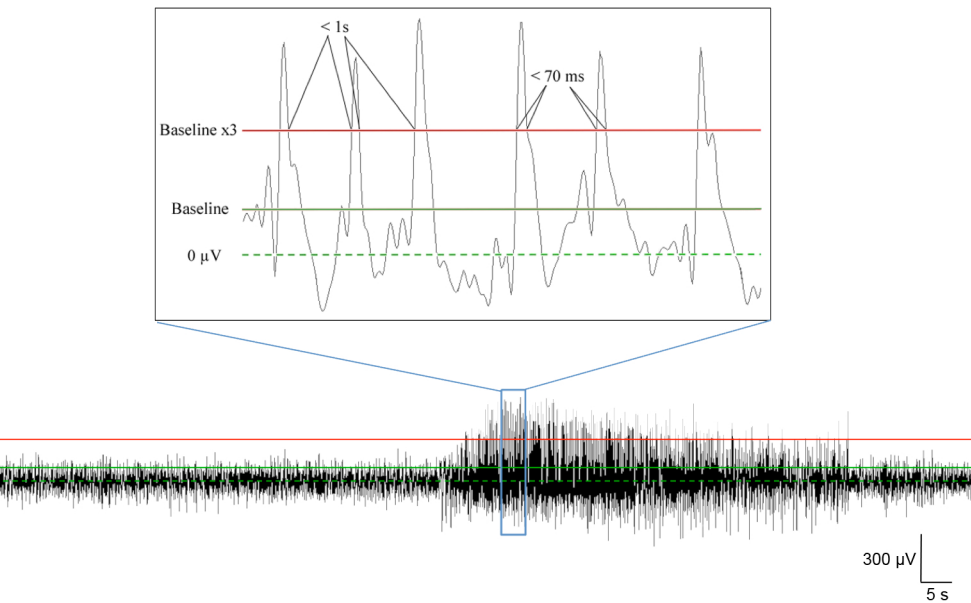

Supplement: Supplementary file 1 — Supporting info item [file BPH-173-1756-s001.pdf]

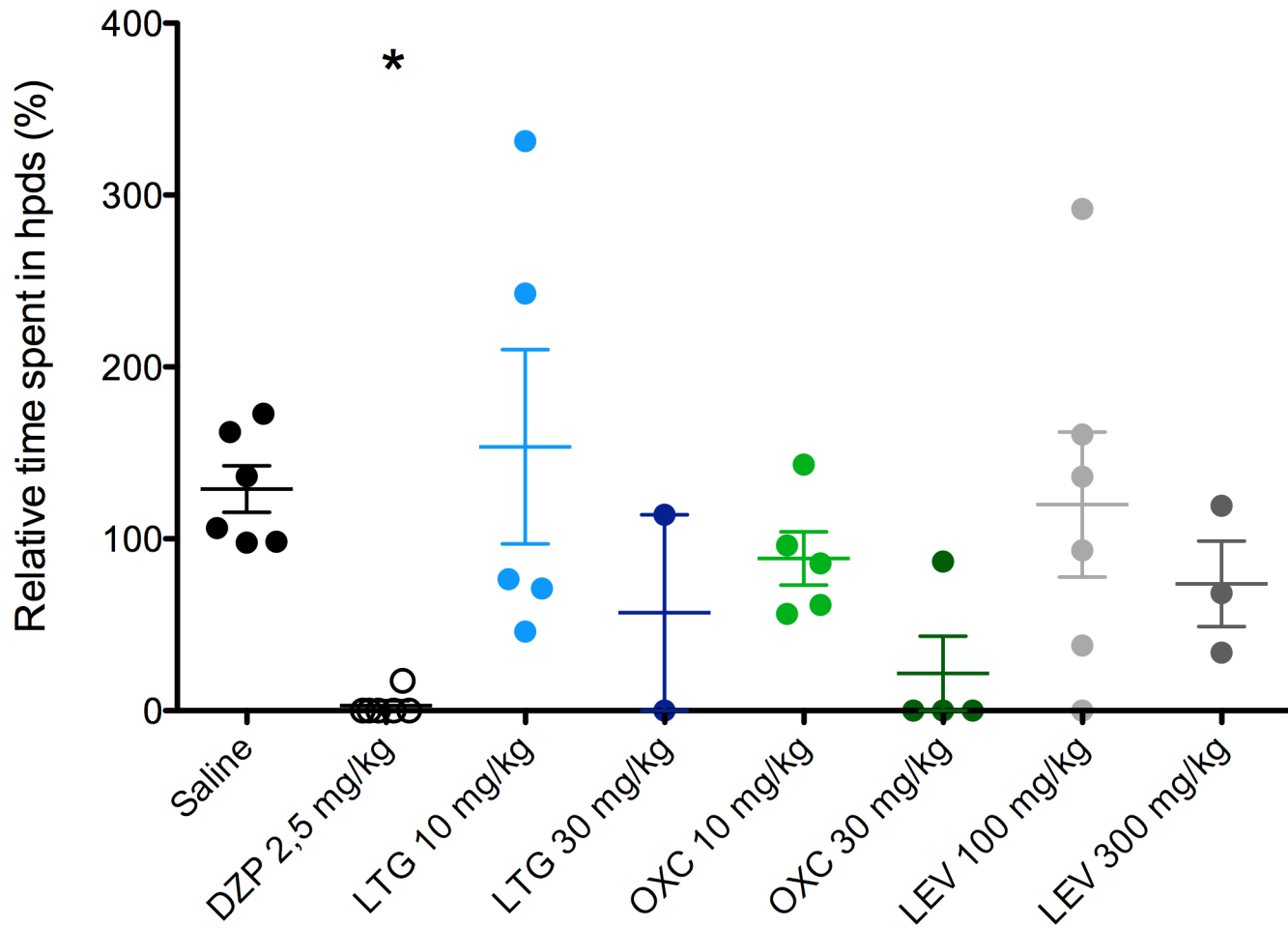

Supplement: Supplementary file 2 — Supporting info item [file BPH-173-1756-s002.pdf]

Saline

U-50488H

6'-GNTI

Specificity control

A

B

C

D

E

F

G

H

Zif 268

pERK 1

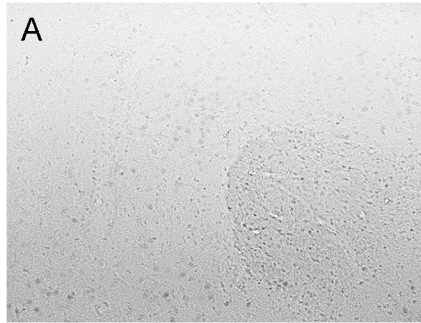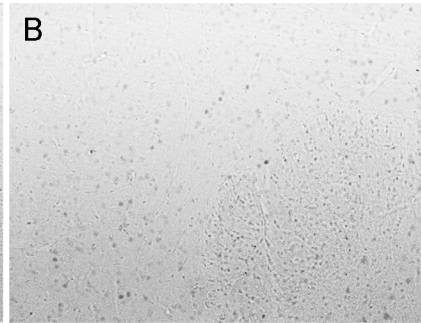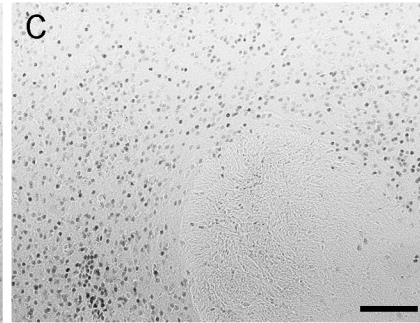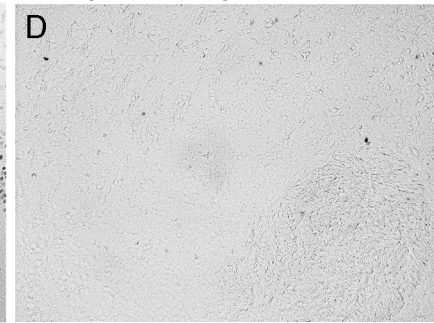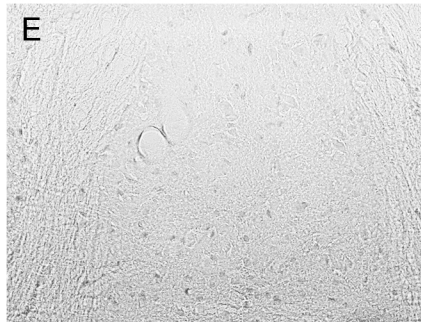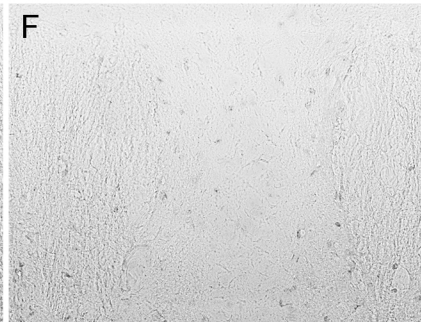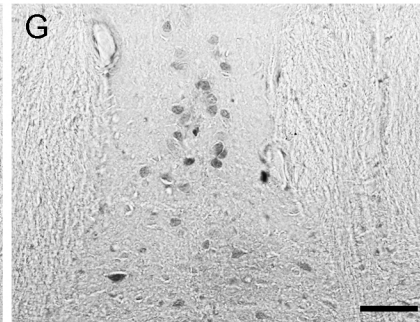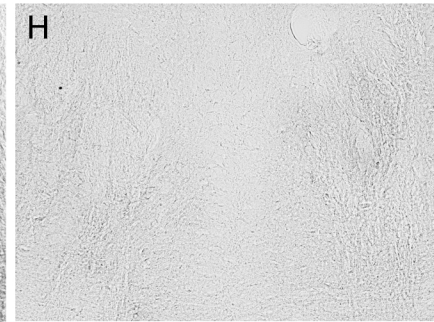

Supplement: Supplementary file 3 — Supporting info item [file BPH-173-1756-s003.pdf]

Relative time of trains (%)

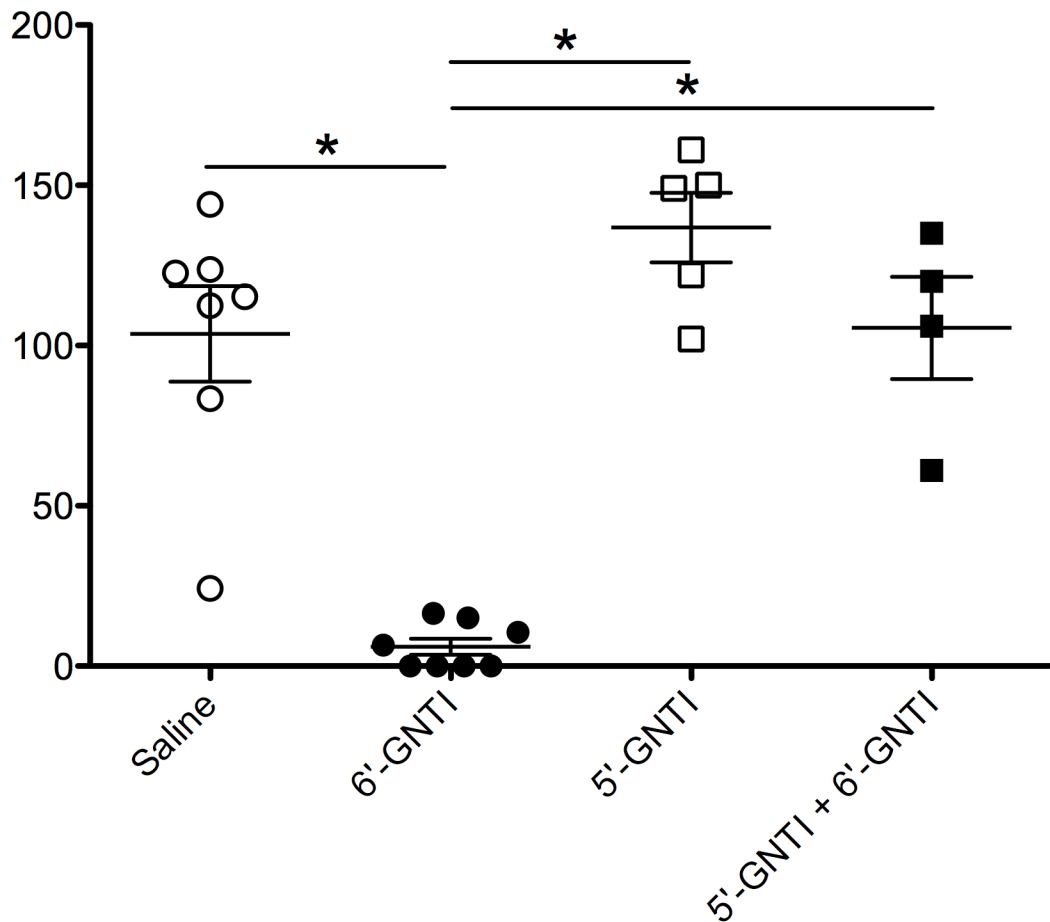

Supplement: Supplementary file 4 — Supporting info item [file BPH-173-1756-s004.pdf]
